# Brief user-controlled admission (BUCA) in psychiatric care

**DOI:** 10.1177/10398562251406034

**Published:** 2025-12-07

**Authors:** Sofie Westling, Josefin Vikström Eckevall, Rose-Marie Lindkvist

**Affiliations:** Psychiatry, Department of Clinical Sciences Malmö, 59568 Lund University, Lund, Sweden; Department of Psychiatry, Skåne University Hospital, Lund, Sweden; Psychiatry, Department of Clinical Sciences Malmö, Lund University, Lund, Sweden; Center for Social and Affective Neuroscience, Department of Biomedical and Clinical Sciences, Linköping University, Linköping, Sweden; Psychiatry, Department of Clinical Sciences Malmö, Lund University, Lund, Sweden

**Keywords:** user involvement, psychiatric, brief admission, self-referral, person-centered care

## Abstract

Brief User-Controlled Admission (BUCA) refers to a set of crisis interventions in which the traditional gatekeeping role of the physician is bypassed, allowing service users to independently decide when to access short-term inpatient care through a pre-negotiated agreement. Examples include Brief Admission by self-referral, Patient-Initiated Brief Admission, Patient-Controlled Admission, and Self-Referral to Inpatient Treatment. The structured agreement promotes predictability and collaboration, enabling the user to assume control over their care. BUCA has been studied in adults and adolescents with severe mental distress at risk for escalation of symptoms, self-harm or suicide. Users report high satisfaction, describing the agreement as a source of psychological safety, dignity, and proactive crisis management. Staff experience strengthened therapeutic alliances and role shifts from gatekeeping to collaboration. Families experience relief, though they may need information and support adapting to the user-led structure. BUCA has been associated with increased functioning in daily life, and lower healthcare costs, particularly among users with high service utilization. While earlier studies have suggested potential reductions in inpatient care, studies including controls have not proven significant effects. Representing a shift towards user-led care, BUCA offers a scalable and potentially cost-effective model aligned with current mental health reform priorities.

## A new paradigm in psychiatric crisis management

Over the past decade, increasing attention has been paid to crisis interventions that emphasize user autonomy and early self-regulation in psychiatric care. Originally developed in the Netherlands,^1^ the model has since been adapted in Sweden as Brief Admission by self-referral (BA; 2–4) and Patient-Initiated Brief Admission (PIBA; 5,6). Both adaptations, designed for individuals with at least three symptoms of borderline personality disorder including recurrent self-harm or suicidality, have been evaluated in adult and adolescent populations.^7–9^ A generic adapted version of Patient-Controlled Admission (PCA) directed at all patients with severe psychiatric conditions has also been implemented and is under evaluation.^10^ Similarly, Patient-Controlled Admission (PCA; 11,12) and Self-referral to Inpatient Treatment (SRIT; 13,14) have been implemented in Denmark and Norway, respectively, allowing users with severe mental disorders to self-refer for short inpatient stays, typically via a prenegotiated agreement.

While these terms—BA, PIBA, PCA, SRIT, and others such as “self-referral admissions,” “green card,” or “contract-based admissions”—describe related practices, the terminology remains inconsistent across jurisdictions, populations, and age groups. To address this, we introduce the term Brief User-Controlled Admission (BUCA) as an overarching concept that encompasses these models while offering a clearer conceptual and clinical framework. Brief User-Controlled Admission (BUCA) refers to psychiatric crisis interventions that enable users with recurring emotional or psychiatric distress to self-initiate short inpatient admissions (typically 1–5 days) based on a pre-established agreement with their care provider. In choosing the term BUCA, “user” replaces “patient” to avoid reducing the person to a receiver of care and instead emphasize their active role in managing their care, consistent with a recovery-oriented and person-centered philosophy.^15^ In this context, the term “user” is employed in the same way as other internationally recognized terms such as “consumer” or “service user.” Likewise, “controlled” replaces “initiated” to stress the user’s autonomy in regulating care. These terminological choices are deliberate, underscoring the ethical and practical significance of user empowerment. BUCA is distinguished not only by providing timely access to care, but through shared responsibility giving the user opportunity to develop and exercise increased control over their healthcare, their situation, their symptoms, and ultimately themselves—supporting self-regulation, dignity, and recovery during periods of instability. Furthermore, BUCA is defined by its predetermined structure, where access is regulated by a prenegotiated agreement outlining limits on duration, frequency, and behavioral expectations.

## Target populations

BUCA has been evaluated in adults and adolescents targeting those with severe mental disorders or behaviors, frequently engaging emergency psychiatric services. Participants include those with repeated self-harm and risk for suicide,^2,5^ personality disorders,^6,11^ affective disorders,^11,13^ psychotic disorders,^11,13^ and anorexia.^16^

Studies from Sweden have shown that users with borderline personality disorder view BUCA as a constructive and empowering alternative to conventional inpatient care, particularly when integrated with ongoing outpatient treatment strategies such as Dialectical Behavior Therapy.^5,6^ The model can be implemented safely also in users with a history of extensive psychiatric inpatient care.^17^

BUCA has been adapted for adolescents meeting criteria for emotional dysregulation and self-harming behavior.^8^ Johansson et al. reported a significant reduction in emergency visits, inpatient days, and coercive care following the implementation of BUCA in adolescent psychiatry. However, a limitation in this study is the lack of control group. These findings are consistent with parental perspectives indicating improved family stability and decreased caregiver burden through BUCA.^9,18^ Lindkvist et al.^7^ further highlighted adolescents’ perceptions of BA as a safe and empowering model of care during crises, noting that adolescents who did not utilize their agreement nevertheless experienced relief and a sense of preparedness by having the option.

## Structure and implementation

BUCA admissions are initiated by users under a formalized written agreement specifying a maximum number of days per admission and a required interval between stays, or a maximum frequency per month. Other non-negotiable parts can be that the user must not put themselves or others at risk during BUCA, or self-harm. If the individual is unable to manage this, they are discharged from the current admission with a referral to appropriate care but retain their agreement and may request a new BUCA when more stable.^3,4^ Usually, the agreement contains individualized parts where the user formulate their own goals, methods to self-regulate or what help can be useful from staff.^3,4^ The agreement thus not only functions as a logistical framework but as a clinical and ethical tool that reinforces user autonomy, structure, and early help-seeking. It clarifies roles and responsibilities, creates predictability, and strengthens the therapeutic alliance by supporting trust and dignity.^19^

The admissions are supported by nursing staff trained in the intervention. Implementation requires careful planning as well as education, financing, restructuring units, quality management, and policy implementation.^20^ Key implementation considerations include availability of beds, coordination across inpatient and outpatient settings, and staff training.^3,4,20,21^

## Clinical effectiveness

Several studies have evaluated the effects of BUCA on service utilization but results have not been consistent. Studies without controls generally report large reductions in psychiatric inpatient care.^8,14,22^ However, all studies including controls have failed to reproduce these findings.^2,11,13^ Thus, the initial results must be interpreted with caution and possibly as an effect of regression toward the mean, given the recruitment of a severely ill population with high health care utilization. However, register-based studies suggest that BUCA can reduce the length of inpatient stays without increasing overall service utilization.^6^ Eckerström et al.^6^ also reported that the BUCA group also had significantly increased outpatient engagement, indicating improved care integration.

With regards to economics, results are also mixed. When using BUCA in units mixed with emergency psychiatric care, BUCA was associated with increased costs in a register-based study with controls.^12^ A health economic evaluation based on a randomized controlled trial took not only the economic part into account, but also health effects.^23^ As compared to treatment as usual, BUCA was found likely to be cost-effective over a 1-year period when added to usual care. BUCA was associated with a significant increase in Quality-Adjusted Life Years (QALY) and, given a comparable bed occupancy rate, not associated with increased costs. Price calculations were based on BA being delivered in a separate unit, while inpatient care days and QALYs were based on BUCA being mixed with emergency care. In long-term follow-ups where also individuals in the control group were offered access to BA, and where BA was provided both in separate and in mixed units in the region where the research took place, patient healthcare costs decreased over a 4-year period^23^ and only a small proportion of those granted access to BUCA continued to use it over time.^24^

An interesting approach was made by Strand and Sjöstrand^16^ who examined if the reallocation of hospital beds from regular admissions to BUCA could be justified, comparing individuals the most severe eating disorders to register controls. They found that self-admission in eating disorder care significantly reduced the need for prolonged hospitalizations and concluded, from a health economics perspective, that reallocating beds to BUCA was justified by the efficiency gains.

## Perspectives from users and loved ones

Qualitative research on user experiences has shown that BUCA was perceived to offer an opportunity for recovery and thereby the possibility to interrupt cycles of worsening symptoms. Being based on trust and availability, BUCA induces a sense of freedom, safety, and active participation among users.^25,26^ Users have reported that knowing an agreement was in place provided psychological safety and reduced anxiety about future crises.^25^ Experienced health care professionals with a warm and attentive approach who could adapt to the individual was expressed as key components.^25,26^ Interviews with relatives and loved ones^26–28^ have pointed towards easy access and trust as key and to the value of a safe back-up for both users and to them as loved ones who are often living with worry. BUCA was experienced to offer users and their relatives hope, fulfilling needs of rest and recovery and enhance relationships, building on personal responsibility. Significant adults to adolescents with access to BUCA described similar experiences but also tension regarding whether responsibility for a severely suicidal and self-harming teenager lies with the healthcare services, the parents, or the adolescent.^29^

## Perspectives from health care professionals

Qualitative research on experiences of BUCA among inpatient and outpatient healthcare professionals has indicated that the intervention complements outpatient treatment, enhances personal development among users and professional development among health care professionals through strengthened person-centered care, structured collaboration, and increased sense of safety.^17,30–34^ Perceived challenges mentioned by health care professionals included experiences of too few beds and working in settings where other types of inpatient care were also provided and the context was not optimally adapted to BUCA.

## Limitations and risks

Not all evaluations of BUCA have shown unequivocal benefits. For example, as described above, Sigrunarson et al.^13^ and Thomsen et al.^11^ found no significant differences in overall service use compared to treatment as usual, highlighting the need for careful integration with broader treatment plans. Paaske et al. found implementation of the model to be more costly.^12^ Furthermore, no controlled studies have shown decreases in self-harm or compulsory admissions, which has been thoroughly investigated.^2,11,13^

Organizational barriers such as bed shortages, staff inconsistency, and lack of clear guidelines have been identified as implementation hurdles.^21^ Unrealistic expectations from users or families about the scope of the intervention may also reduce satisfaction if not properly addressed.^35^ Concerns have also been raised about displacement effects, but no clear evidence of this has been observed in the studies published to date.^16^

## Implications for practice

BUCA has primarily been implemented in Northern Europe, however, given the growing demand for flexible, user-centered models of psychiatric care, it holds promise for adoption in health systems across other continents. Its emphasis on early intervention, respect, and shared responsibility aligns with current mental health policy goals in many regions. However, successful implementation will require:

(1) Adaptation to local legal and cultural contexts
(2) Designation of specific beds and trained staff
(3) Structured evaluation of outcomes and fidelity to the model

Collaborative planning with users, caregivers, and multidisciplinary teams will be essential to tailoring BUCA to diverse care environments. Furthermore, clear guidelines on liability as well as legal frameworks, are essential for safe implementation, as this model entails a shift from control and responsibility outside traditional frameworks.

## Conclusion

BUCA represents a paradigm shift in psychiatric crisis intervention, reframing inpatient care as a preventive, user-led tool. While evidence from Scandinavia supports its efficacy in reducing acute distress, enhancing autonomy, and improving service satisfaction, further research and culturally sensitive implementation strategies are needed for broader implementation.

## Data Availability

Not applicable, as this is a clinical update that only includes a summary of previously published studies.*
